# Supplementary figures and images for: Identification of potent anti-Cryptosporidium new drug leads by screening traditional Chinese medicines
Source: PLoS Negl Trop Dis. 2022 Nov 28;16(11):e0010947. doi: 10.1371/journal.pntd.0010947 (PMC9731497; doi:10.1371/journal.pntd.0010947)

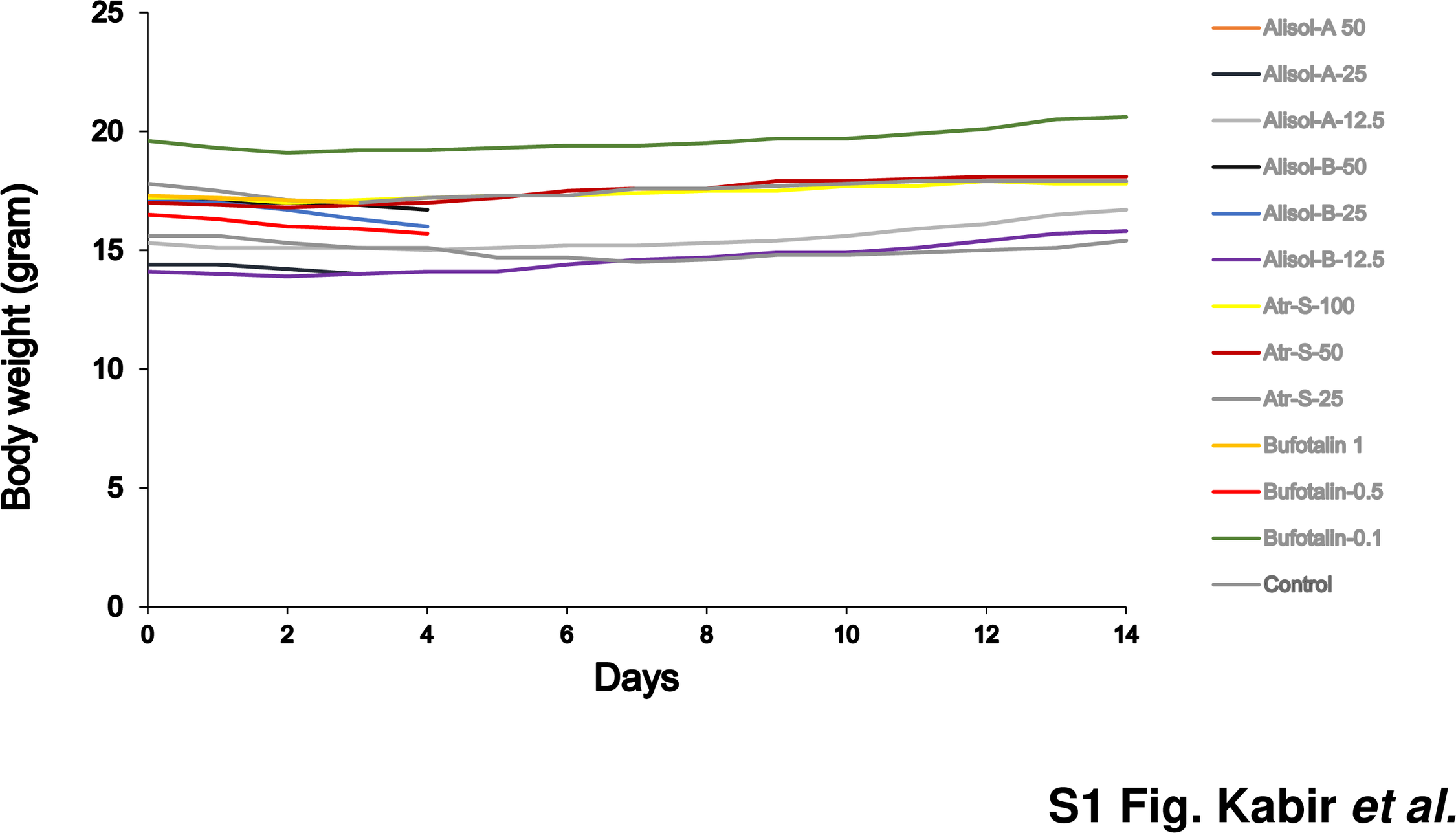

Supplement: S1 Fig — The body weights of mice infected with C. parvum are shown with the following drugs. Control: Infected-untreated mice, Alisol-A: Infected mice treated with different concentration of alisol-A at 50, 25, and 12.5 mg/kg for 11 consecutive days, Alisol-B: Infected mice treated with different concentration of alisol-B at 50, 25, and 12.5 mg/kg for 11 consecutive days, Atr-S: Infected mice treated with different concentration of atropine-sulfate at 100, 50, 25mg/kg for 11 consecutive days, Bufotalin: Infected mice treated with different concentration of bufotalin at 1, 0.5, and 0.1 mg/kg for 11 consecutive days. Treated mice values were not significant compared with those of uninfected mice. (TIF) [file pntd.0010947.s001.tif]

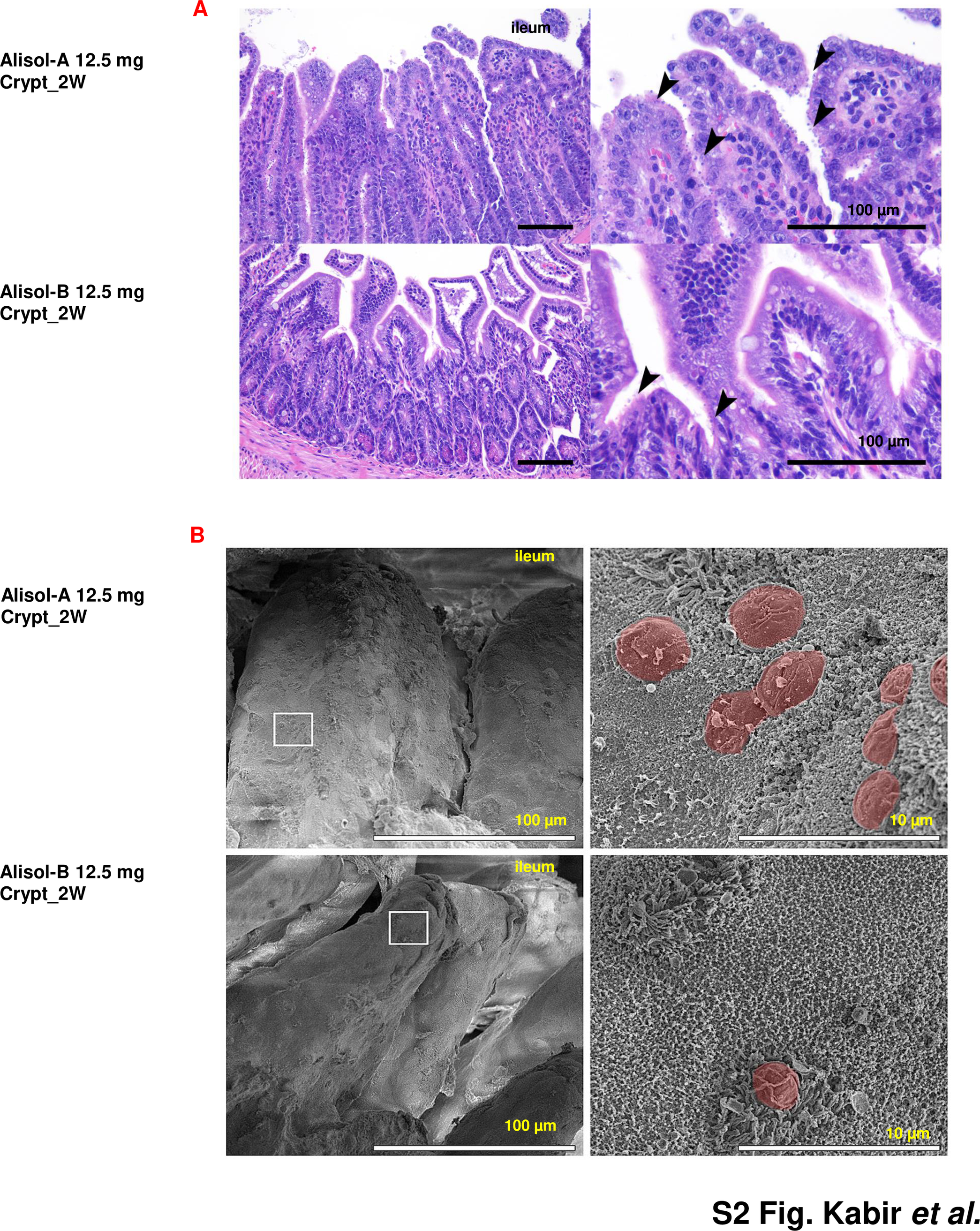

Supplement: S2 Fig — (A) Histological sections of the small intestine (ileum) of different animal groups (treated with alisol-A and alisol-B). All mice were orally inoculated with Cryptosporidium parvum oocyst (1×10^5^), then treated with each compound. The severity of C. parvum infection was scored from–to 3+. Ileal sections of treated groups [Alisol-A (Score: 3+) and Alisol-B (Score: 2+)] showing no remarkable reduction in the number of oocysts (arrowheads: C. parvum oocysts). HE. Bar = 100 μm. (B) Scanning electron microscopic (SEM) images of intestinal ileum tissue of two groups of mice infected with C. parvum. The SEM shows the intracellular structures as well as the surface of C. parvum attached to the host cells. (TIF) [file pntd.0010947.s002.tif]

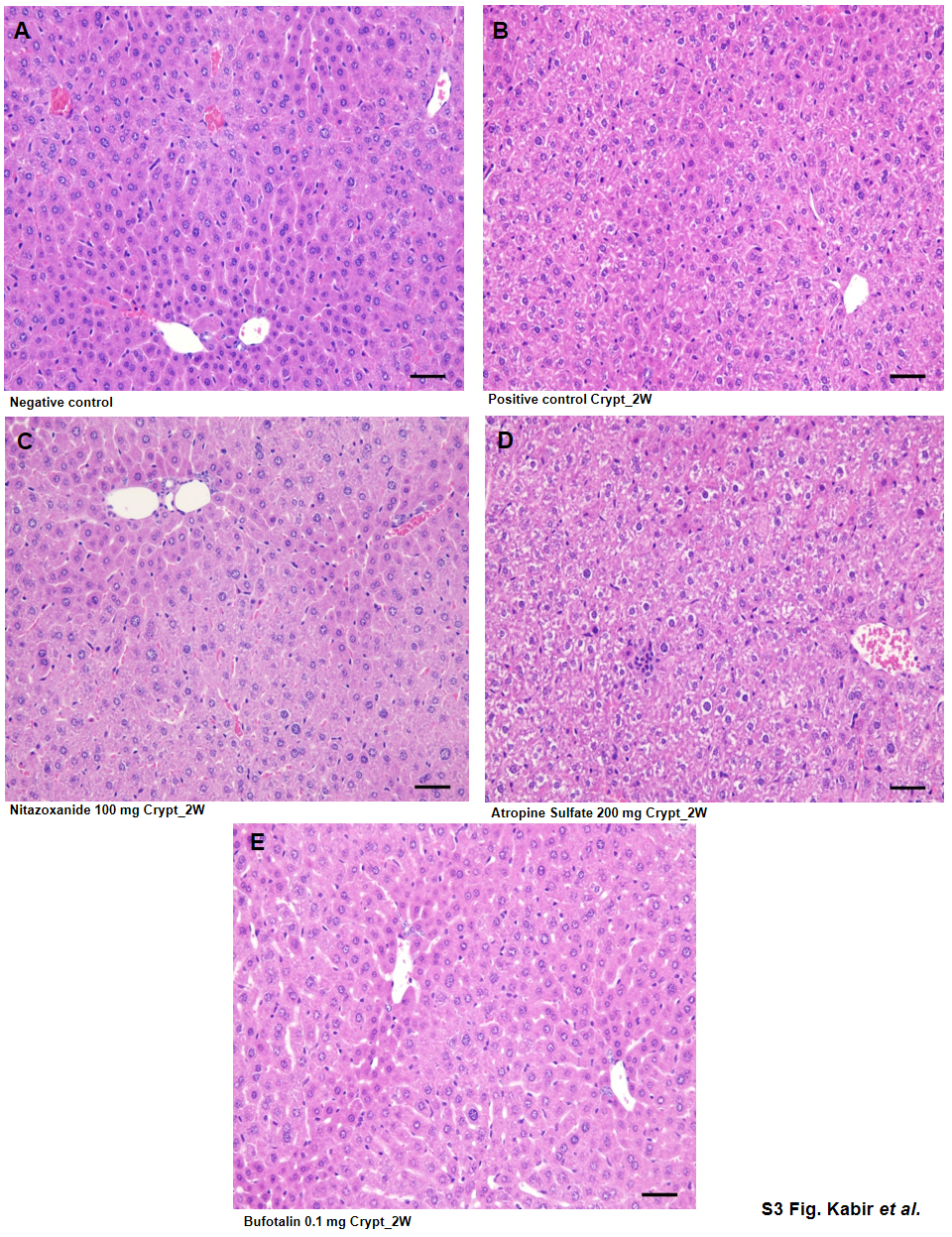

Supplement: S3 Fig — The higher magnification for the histological sections of the liver of different animal groups. Except for the negative control group, all mice were orally inoculated with Cryptosporidium parvum oocysts (1×10^5^), then treated with each compound. (A) Uninfected mouse (negative control) showing karyomegaly and hepatocellular hypertrophy. (B) Non-treated mouse (positive control) showing hepatocellular hypertrophy and inflammation of the hepatocytes. (C) Nitazoxanide-treated groups showing karyomegaly and hepatocellular hypertrophy. (D) Atropine sulfate-treated group showing inflammation. (E) Bufotalin-treated group showing also inflammation and hepatocellular hypertrophy. Hematoxylin and eosin (HE) stain, Bar = 50 μm. (TIF) [file pntd.0010947.s003.tif]
