# Supplementary material for: Identification of potent anti-Cryptosporidium new drug leads by screening traditional Chinese medicines
Source: PLoS Negl Trop Dis. 2022 Nov 28;16(11):e0010947. doi: 10.1371/journal.pntd.0010947 (PMC9731497; doi:10.1371/journal.pntd.0010947)
Supplement: S1 Table — (DOCX) [file pntd.0010947.s004.docx]

**Supporting information**

**S1 Table.** Comparative efficacies of four hit compounds treated for reducing oocysts shedding of *C. parvum*-infected neonatal SCID mice.

| **Days of treatment** | **Treatment compounds with dosages** | **No. of mice** | **% of oocysts shedding** | **% reduction in oocysts shedding** |
| --- | --- | --- | --- | --- |
| 11 | Alisol-A-50mg/kg BW | 1 | ** | ** |
| 11 | Alisol-A-25mg/kg BW | 1 | ** | ** |
| 11 | Alisol-A-12.5mg/kg BW | 1 | 55.6 | 44.4 |
| 11 | Alisol-B-50mg/kg BW | 1 | ** | ** |
| 11 | Alisol-B-25mg/kg BW | 1 | ** | ** |
| 11 | Alisol-B-12.5mg/kg BW | 1 | 61.3 | 38.7 |
| 11 | Atropine sulfate-100mg/kg BW | 1 | 45.1 | 54.9 |
| 11 | Atropine sulfate-50mg/kg BW | 1 | 49.1 | 50.9 |
| 11 | Atropine sulfate-25mg/kg BW | 1 | 75.2 | 24.8 |
| 11 | Bufotalin-1mg/kg BW | 1 | ** | ** |
| 11 | Bufotalin-0.5mg/kg BW | 1 | ** | ** |
| 11 | Bufotalin-0.1mg/kg BW | 1 | 28.8 | 71.2 |
|  | Control* | 1 | 100 | 0.0 |

* Untreated mice infected with HNJ-1 strain of *C. parvum* oocysts.

** Unable to figure out the percentage of oocysts discharged due to the mortality of mouse.
