# Supplementary material for: Identification of potent anti-Cryptosporidium new drug leads by screening traditional Chinese medicines
Source: PLoS Negl Trop Dis. 2022 Nov 28;16(11):e0010947. doi: 10.1371/journal.pntd.0010947 (PMC9731497; doi:10.1371/journal.pntd.0010947)
Supplement: Attachment — Submitted filename: Response to Reviewers.docx [file pntd.0010947.s006.docx]

**Reviewer comments and author responses**

Editor in Chief

We would like to express our greatest thanks to the editors and all the reviewers for their effort to evaluate our original manuscript thoroughly, and their objective suggestions and comments. We have reviewed our manuscript taking into account all the suggestions and comments. The revised parts in the manuscript are indicated in red, and the changes in the revised text are summarized in our response below. The newly added parts for the point-by-point responses to the reviewers’ comments are underlined.

**Point-by-point responses to the Reviewer #1:**

**Results**

**Response to Reviewer #1**: -Does the analysis presented match the analysis plan? Mostly YES

-Are the results clearly and completely presented? NO

-Are the figures (Tables, Images) of sufficient quality for clarity? YES

**Response:** Thank you for your comment.

**Editorial and Data Presentation Modifications?**

**Reviewer #1**: It is clear the authors first language is not English, it is not the worst written manuscript I have read but would benefit from someone reading and correcting the grammar prior to submission.

**Response:** We appreciate your comment. We already checked grammar in our manuscript by native English speaker, who got Ph.D degree in microbiology. We paid her for English editing before the submission.

**Summary and General Comments**

**Reviewer #1**: The authors determine the effect of a series of compounds used in traditional Chinese medicine on the in vitro and in vivo growth of Cryptosporidium parvum. The results indicate the potential of bufotalin and possibly atropine sulfate for the chemotherapy of Cryptosporidosis, which currently lacks a robust chemotherapeutic strategy. Overall, the data is interesting and would be a useful guide to researchers in the field potentially leading to new chemotherapies for this neglected disease. In it’s present form there are many inconsistencies that should be addressed; some of these are grammatical and are easy fixes, but there are also some technical problems in the methodology that should be addressed prior to publication.

**Response:** Thank you for your comments.

**Specific comments:**

1. Summary–line 46 change ‘top goal’ to priority

**Response:**

Thank you for this suggestion. We changed and revised this word in line 47, in our revised manuscript.

2. Summary–line 54 replace ‘also showed in vivo inhibitory effects’ with reduced oocyst shedding by 68% and 78%, respectively.

**Response:**

Thank you for this suggestion. We replaced and revised this word in line 54, in our revised manuscript.

3.      Summary – line 56-57 Could be good candidates … sounds as if the authors are uncertain of the potential better to state: The chemotherapeutic potential of these compounds are discussed.

**Response:**

Thank you for this suggestion. We replaced and revised this word in line 57, in our revised manuscript.

4.      Introduction – line 70 should add or children under 2 years after immunodeficient patients.

**Response:**

Thank you for this suggestion. We added this word in line 71, in our revised manuscript.

5.      Methods – line 131 delete ‘To begin’

**Response:**

Thank you for this suggestion. We deleted this word in line 131, in our revised manuscript.

6.      Methods – line 139 the composition and concentration of phosphate buffer should be stated.

**Response:**

Thank you for this suggestion. We modified and added the composition and concentration of phosphate buffer in line 139, in our revised manuscript.

0.1 M phosphate buffer (with 93.4 mM K_2_HPO_4_ and 6.5 mM KH_2_PO_4_; pH 8.0).

7.      Methods - line 140 similarly the composition and concentration of phosphate buffered saline should be stated, and the filter conditions described – what pore-size filter, syringe or pump driven, etc.

**Response:**

Thank you for this suggestion. We modified and added the composition and concentration of phosphate buffered saline, and the filter conditions described in line 140-142, in our revised manuscript.

The oocyst/sporozoite suspensions were then rinsed in 1x PBS (with 137 mM NaCl, 2.7 mM KCl, 8 mM Na_2_HPO_4_, and 2 mM KH_2_PO_4_; pH 7.5) once more before being filtered through a 5-μm pore-size PVDF filter (Millipore, Burlington, MA, USA) with 5 ml syringe to remove the oocyst wall.

8.      Methods – line 141 the HCT-8 cells were ‘treated’ with C. parvum? Do they mean ‘infected’

**Response:**

Thank you for this suggestion. Yes, infected. We replaced and revised this sentence in line 143-145, in our revised manuscript.

HCT-8 cells were seeded in 96-well plates (Thermo Fisher Scientific Inc., MA, USA) and incubated overnight. The cells were then infected with *C. parvum* sporozoites (4 ×10^4^ sporozoites/well) containing in RPMI-1640 medium for 3 h.

9.      Methods – line 142 are the compounds in the 1 uM library dissolved in DMSO? The statement ‘uninvaded parasites’ is misleading and should be changed – the host cells are invaded?

**Response:**

Thank you for your comments. We mentioned in line 108, in our original manuscript that the compounds were dissolved in DMSO. This is our stock volume. But at the time of working we made our desired concentration of the compounds dissolved in RPMI-1640 medium. Actually, wash out cannot completely remove uninvaded parasite (McCowin et al., 2022). To prevent confusion of the reader, we change “uninvaded parasite” to “non-infected parasite or debris”. We modified this sentence and added in line 145-146, in our revised manuscript.

Ref:

McCowin S, Petri WA Jr, Marie C. Protein Kinase C- α is a gatekeeper of *Cryptosporidium* sporozoite adherence and invasion. Infect Immun. 2022; 90(3):e0067921.

After 3h inoculation, the non-infected parasite or debris were washed with new RPMI-1640 media. At this point, in RPMI-1640 medium containing 1 μM library compounds were added.

10.     Methods – line 144 the description sounds like the compounds added twice? Is this correct.

**Response:**

Thank you for your comment. We didn’t use the compounds twice. We omitted this word and added sentence as we mentioned above in line 146-147, in our revised manuscript.

11.     Methods – line 145-147 The fixation step needs more details, is the medium removed prior to addition of methanol? Also the statement ‘the cells were blocked…’  is vague, what cells host, parasite? And Sporo-Glo primarily stains sporozoites that are extracellular, motile stages, which are presumably removed by the washing? Is this method staining intracellular meront stages if so an explanation is needed.

**Response:**

Thank you for your comments. Yes, the culture medium were gently removed, then performed methanol fixation. The parasite-infected host cells were then blocked. Immunofluorescence blocking was performed by using 1% bovine serum albumin (BSA) in PBS for 30 minutes and then stained with Sporo-Glo. Sporo-Glo can stain all the stages of *Cryptosporidium;* sporozoites, trophozoite, meront (Boxell et al., 2008). Washing before methanol fixation was performed extracellular parasite (non-infected parasite or debris) to remove as previously I answered in your comment 9. The ice-cold 100% methanol has an action of cell permeabilization. So, both sporozoites and meront were stained regardless of intra-or extra-cellular parasite but it is known that sporozoites are differentiated into trophozoite within few hour post infection. Therefore, sporozoites were not exist in 48 hours of post infection (Jumani et al., 2019). In this study, we count a vacuole as one parasite, even if the vacuole contains a number of trophozoites and meronts. Taken together, in this study, we counted intracellular infected parasites. We modified the fixation step in details in line 148-156, in our revised manuscript.

Following incubation, the infected cells were fixed for 10 minutes in ice-cold 100% methanol at room temperature. Plates were then carefully rinsed 3 times with PBS. The parasite infected cells were then blocked with 1% bovine serum albumin (BSA) in PBS for 30 minutes and then stained with Sporo-Glo (an anti-Cryptosporidium polyclonal antibody) (Waterborne Environmental, Inc., VA, USA) protected from light for 1 h at room temperature. Finally, cells were washed twice with 1 x PBS-T (1 x PBS supplemented with 0.01% Tween). A Keyence BZ-II 9000 was used to capture the images (KEYENCE, Osaka, Japan). The plates were imaged with a HS all-in-one fluorescence microscope using a 20 x objective. The image output was imported into Microsoft excel for data organization and analysis. All C. parvum parasite in the well were counted.

Ref:

Boxell A, Hijjawi N, Monis P, Ryan U. [Comparison of various staining methods for the detection of Cryptosporidium in cell-free culture.](https://pubmed.ncbi.nlm.nih.gov/18547565/) Exp Parasitol. 2008; 120(1):67-72.

Jumani RS, Hasan MM, Stebbins EE, Donnelly L, Miller P, Klopfer C, et al. [A suite of phenotypic assays to ensure pipeline diversity when prioritizing drug-like *Cryptosporidium* growth inhibitors.](https://pubmed.ncbi.nlm.nih.gov/31015448/) Nat Commun. 2019; 10(1):1862.

12.     Methods line – 180. What are the C. parvum oocysts suspended in?

**Response:**

The *C*. *parvum* oocysts were suspended in cold, sterile, distilled water.

13.     Methods line – 185. What unfavorable effects?

**Response:**

The unfavorable effects means no obvious health effects were found. For example, the mice body weight, fur condition, posture, and lethargy. If this word makes misleading, we removed this word in line 191, in our revised manuscript.

14.     Methods line – 193-195. In mouse model infections oocyst shedding does not occur under 5 days? How were the oocysts detected, using a modified acid-fast stain, Crypt-a-Glo?

**Response:**

Thank you for your comments. We agree with your concern. In general, we can observe *Cryptosporidium* oocysts shedding from 5 days of post infection in mouse model. Often we can observe the differences of the treated groups due to the drug treatment. So, we decided to collect the data from the date of drug induced. However, [Chai et al. (1999)](https://www.sciencedirect.com/science/article/pii/S2211320721000452#bib19) found that oocyst excretion started on the 4th day PI in both immunocompetent and immunosuppressed mice. Moreover, another study found that oocysts shedding after 2 days of post infection in mouse model (Mammeri et al., 2018). We detected oocysts by microscopic method using sucrose floatation method.

**Ref:**

Chai JY, Guk SM, Han HK, Yun CK. **Role of intraepithelial lymphocytes in mucosal immune responses of mice experimentally infected with** Cryptosporidium parvum. J. Parasitol. 1999; 85(2):234-9.

Mammeri M, Chevillot A, Thomas M, PolackB, Julien C, Marden JP, et al. [Efficacy of chitosan, a natural polysaccharide, against *Cryptosporidium parvum* in vitro and in vivo in neonatal mice.](https://pubmed.ncbi.nlm.nih.gov/30237052/) Exp Parasitol. 2018; 194:1-8.

15.     Methods line – 199 side effects should be changed to toxicity.

**Response:**

Thank you for your suggestion. We changed this word in line 206, in our revised manuscript.

16.     Methods line – 210-212. The opening statement to this section indicates evaluation of growth inhibition and toxicity bit it is not clear how will toxicity be evaluated using this method?

**Response:**

Thank you for your comment. Histopathological examination is classical and effective method for screening of *in vivo* effects of new medical compounds. *In vivo*, we examined multiple organs (liver, heart, lung, kidney and intestines) and evaluated the growth inhibitory effect and toxicity for host tissue by histopathologist.

17.     Methods paragraph starting line – 214. A statement indicating what structural features are being looked at to evaluate toxicity should be added.

**Response:**

Thank you for your comment. Sub-effects of drugs (hepatocellular damage) were evaluated for major histological changes in karyomegaly, hepatocellular hypertrophy, and interstitial fibrosis/inflammation. We also checked the organs (liver, heart, lung, kidney, and intestines) alteration at necropsy whether it has abnormal or normal. We added this sentence in methods in line 219-222, in our revised manuscript. Some histological findings (karyomegaly, hepatocellular hypertrophy, or interstitial inflammation) were observed in all examined livers (S3 Fig), it suspected sub-effect of compounds or infection, but there was no difference in distribution and severity between experimental groups. We added this sentence in results in line 295-298, and in the supporting information line 609-617, in our revised manuscript.

18.     Results – why did the authors double the dose for atropine from 100 to 200 mg/kg when it is clear that 100 mg/kg did not cure? This is especially relevant since bufotalin was used at a fraction of this dose 0.1-0.025 mg/kg. With a mouse dose of 200 mg/kg it would scale to >10 g to treat a human?

**Response:**

We appreciate your comments. We induced double the dose of atropine from 100 to 200 mg/kg because of two reasons; one that the lethal dose (LD_50_) of drug orally for mice is higher, and another there was no mortality found in the previous 100 mg/kg dose and we expected to cure. We think even though it is a high dose, it can be improved by further adjusting because it is only a newly found lead compound. For bufotalin, we found better response in our earlier trial using the fraction of this dose (1, 0.5, 0.1 mg/kg). Then we used lower fraction (0.1-0.025 mg/kg) for retaining this better response by using a lower dose of drug. In addition to this, the LD_50_ of this drug is lower. Previously, one report mentioned the conversion of mouse dose of 200 mg/kg would scale to 16.2 mg/kg for human treatment (Nair and Jacob, 2016).

Ref:

Nair AB, Jacob S. A simple practice guide for dose conversion between animals and human. J Basic Clin Pharm. 2016; 7(2):27-31.

19.     Results – The data in Table 2 should be at best to one decimal place, the confidence in two decimal places is not supported by the analysis used.

**Response:**

Thank you for your comment. We modified and put one decimal in Table 2 in our revised manuscript.

20.     Results – since the in vitro method used to evaluate parasites fixes them, the authors do not know if what they detect are non-viable stages which can give misleading data. Do the authors know if parasites can recover after treatment, this could have been done by taking a sample from in vitro incubation with the compound for a fixed time and infecting mice? Alternatively using RT-PCR of parasite 18-S RNA which has a short half-life would be a better indicator of viable parasite load. The uncertainty of parasite viability post compound treatment might explain some of the differences between in vitro and in vivo data obtained.

**Response:**

Thank you for your comments. We don’t know whether this method is an effective or not in the *Cryptosporidium* assay, however, we think this might be a kind of strategy. But we are sorry to say that we didn’t focus this strategy. We can think it in our future plan.

**Point-by-point responses to the Reviewer #2:**

**Methods**
**Reviewer #2:** 1) objectives are clear

2)  study design is appropriate

3)  sample size may be too small (3 mice per group in final efficacy study)

4)  one-way ANOVA with correction was used and is appropriate--they need to show the statistics in table 3 in addition to stars in figure 4

5)  no ethical concerns

**Response:**

Thank you for your comment. We added the information of statistics in Table 3. The nature of the error bars in Figure 4 indicated in the figure legend in line 534, in our revised manuscript.

**Results**

**Reviewer #2:** 1)  analysis presented is appropriate

2)  results are clearly presented but the authors have not yet made all data available, which they state will be done upon acceptance.  More information re statistics is needed in Table 3.  The nature of the error bars in Figure 4 needs to be indicated in the figure legend.

3)  the figures and tables are clear--as above, clarifications/additions are needed for table 3 and figure 4.

**Response:**

Thank you for your comment. We added the information of statistics in Table 3. The nature of the error bars in Figure 4 indicated in the figure legend in line 534, in our revised manuscript.

Pairwise comparisons using a one-way ANOVA with the post-hoc Tukey HSD test were used to determine whether reductions in numbers of oocysts for treated vs. untreated control mice were statistically significant. Results were considered to be significant for *P < 0.05 or **P < 0.01.

**Editorial and Data Presentation Modifications?**

**Reviewer #2:** Editorial suggestions:

1. Candidates is an overstatement, as implies optimization and derisking have been completed.  More appropriate drug leads--same in abstract and throughout the manuscript

**Response:**
We appreciate your comment. We replaced and revised this word in our abstract and throughout the revised manuscript.

2. Line 75 about clofazimine is out of date.  This repurposing screening hit was tested in a clinical trial in HIV patients with cryptosporidiosis and it was ineffective (see Clinical Infect Dis. 2021. 73: 183-91 and Clinical Infect Dis. 2021. 73: 192-4.)

**Response:**
We appreciate your comment. We agree with your comment, but recently a published research reveal to make explore whether lack of efficacy of clofazimine. The findings suggested clofazimine will provide a remedy for the cryptosporidiosis patients currently without a viable treatment option (Zhang et al., 2022).

Ref:

Zhang CX, Love MS, McNamara CW, Chi V, Woods AK, Joseph S, et al. [Pharmacokinetics and Pharmacodynamics of Clofazimine for Treatment of Cryptosporidiosis.](https://pubmed.ncbi.nlm.nih.gov/34748385/) Antimicrob Agents Chemother. 2022; 66(1):e0156021.

3.  Line 82 indicating a lack of studies showing compounds in cattle that reduce clinical symptoms is incorrect.  Many studies have now been published for compounds in development showing efficacy against diarrhea in dairy calves (see PMID 31249291, PMID 29309415, PMID 28562588, and PMID 27923949).

**Response:**

Thank you for your comment. We agree with your comment, we modified and revised this sentence in line 80-83, in our revised manuscript.

Several additional chemotherapeutic drugs have been studied in livestock for the treatment of cryptosporidiosis showing efficacy against diarrhea in dairy calves [11-13], however, we need more screening to develop and identify new drug lead compounds to combat this infectious pathogen.

Ref:

11. Stebbins E, Jumani RS, Klopfer C, Barlow J, Miller P, Campbell MA, et al. [Clinical and microbiologic efficacy of the piperazine-based drug lead MMV665917 in the dairy calf cryptosporidiosis model.](https://pubmed.ncbi.nlm.nih.gov/29309415/) PLoS Negl Trop Dis. 2018; 12(1):e0006183.

12. Lunde CS, Stebbins EE, Jumani RS, Hasan MM, Miller P, Barlow J, et al. [Identification of a potent benzoxaborole drug candidate for treating cryptosporidiosis.](https://pubmed.ncbi.nlm.nih.gov/31249291/) Nat Commun. 2019. 10(1):2816.

13. Schaefer DA, Betzer DP, Smith KD, Millman ZG, Michalski HC, Menchaca SE, et al. [Novel Bumped Kinase Inhibitors Are Safe and Effective Therapeutics in the Calf Clinical Model for Cryptosporidiosis.](https://pubmed.ncbi.nlm.nih.gov/27923949/) J Infect Dis. 2016; 214(12):1856-1864.

4.  Line 134--use of nitazoxanide as comparative drug.  Specify what concentration of nitazoxanide was used for this control.  Was it 1 micromolar like the screening compounds?  Nitazoxanide lacks potency and is inactive at 1 micromolar in our hands and also published. literature.

**Response:**

Thank you for your comments. We were used infected HCT-8 cells without drug and uninfected HCT-8 cells without drug as positive and negative controls, respectively, mentioned in line 133-134, in our original manuscript. We used 10 μM concentration of nitazoxanide. We mentioned that nitazoxanide has an IC_50_ against *C. parvum* of ~3.7 μM (Bessoff et al., 2013) in line 320-321, in our original manuscript. We added this concentration in the figure legends in line 508, in our revised manuscript.

5.  Figure 4:  please specify what the error bars are in the figure legend.  Are these standard deviation or standard error?

**Response:**
Thank you for your comment. These are the standard error in line graph. Line graph data are the mean oocyst counts and standard error of the mean (SEM) (n=3 mice per group). We added this information to specify the error bars in the figure legend in line 534, in our revised manuscript.

Data shown are the mean and SEM (n = 3 mice in each group).

6.  Table 3:  a column should be added or asterisks, etc and a definition to indicate the results of statistical analysis.  Also, for calculation of percent inhibition, are these comparisons vs control for a single time point or for area under the curves shown in figure 4?

**Response:**

Thank you for your comments. We rechecked the analysis and are sorry for the mistake the data of 95% CI. We added the corrected 95% CI data and indication of statistical analysis in Table 3 in line 578-579, in our revised manuscript. Yes, the percent inhibition is the comparison of control group vs. individual treated groups in consideration of the area under the curves shown in figure 4.

Microsoft excel was used to calculate the percent inhibition of infection as follows: 1-(mean number of parasites in drug treated group/mean number of parasites in control group) X 100. Pairwise comparisons using a one-way ANOVA with the post-hoc Tukey HSD test were used to determine whether reductions in numbers of oocysts for treated vs. untreated control mice were statistically significant. Results were considered to be significant for *P < 0.05 or **P < 0.01.

7.  Discussion lines 306-309:  It is surprising and a little concern that there is no overlap in the hits vs Plasmodium and Toxoplasma, given the related of these species with Cryptosporidium.  While differences in PK/PD requirements for in vivo efficacy can easily account for in vivo differences, most studies show an enrichment of active compounds in vitro (e.g. screens that have been done using the MMV Malaria Box; PMID 24566188 and PMID 29339392).  More discussion of this would be of interest.

**Response:**

Thank you for your critical comments. We agree with your statement, however, in this study there is no overlap in the hits vs Plasmodium and Toxoplasma. The previous studies were not focus on the mechanism of function of compounds that inhibited the parasite growth and there were no more research findings of these TCMs compounds against apicomplexan parasites. We are also wondering that alisol-A and alisol-B has anti-*Plasmodium* activity reported previously (Adams et al., 2011). We added the probable reason in line 324-327, in our revised manuscript.

It is surprising that there is no overlap in the hits vs. *Plasmodium* and *Toxoplasma*, given the related of these species with *Cryptosporidium*. It might be due to the different enzyme related to metabolic cyclic pathways or functions related to either host or parasite factors.

Ref:

Adams M, Gschwind S, Zimmermann S, Kaiser M, Hamburger M. [Renaissance remedies: Antiplasmodial protostane triterpenoids from Alisma plantago-aquatica L. (Alismataceae).](https://pubmed.ncbi.nlm.nih.gov/21354292/) J Ethnopharmacol. 2011; 135(1):43-7.

8.  Line 327:  lack of in vivo efficacy for Alisol-A and -B.  This discussion needs to be expanded some.  There is considerable data at this point suggesting that in vivo efficacy vs. Cryptosporidium is dependent on prolonged intestinal exposure.  Lack of adequate intestinal exposure is the likely explanation (see PMID 28541457).  This is important because it may be that modifications of Alisol-A and -B would produce compounds with better intestinal exposure and in vivo efficacy.

**Response:**

Thank you for this comment. We agree with your concern. Lack of adequate intestinal exposure is one of the factors. However, sometimes it depends on the other factors like the nature of chemical compounds, dose of compounds, the age and which type of mice used. We modified our explanation as you suggested and added in line 343-348, in our revised manuscript.

One of the factors might be the lack of adequate intestinal exposure to these drugs. Because it is likely that prolonged intestinal exposure of these TCMs is crucial for the efficacy in vivo. This is important because it may be that modifications of alisol-A and alisol-B would produce compounds with better intestinal exposure and *in vivo* efficacy. The nature of the chemical compounds, whether the drug compounds are permeable or less permeable to the small intestine would be the other factors [31].

Ref:

31. Arnold SLM, Choi R, Hulverson MA, Schaefer DA, Vinayak S, Vidadala RSR, et al. [Necessity of Bumped Kinase Inhibitor Gastrointestinal Exposure in Treating *Cryptosporidium* Infection.](https://pubmed.ncbi.nlm.nih.gov/28541457/) J Infect Dis. 2017; 216(1):55-63.

9.  Line 341 re mechansim of nitazoxanide:  While nitazoxanide is a PFOR inhibitor, there is considerable evidence that that is not its mechanism of action against Cryptosporidium species.  Cryptosporidium species have an alternative PFOR that nitazoxanide and more potent analogs don't inhibit (see PMID 30297368).

**Response:**

Thank you for this suggestion. We agree with your concern. We modified this sentences in line 361-363, in our revised manuscript.

Most believe that nitazoxanide anticryptosporidial effect is off-target, as *Cryptosporidium* species has an alternative PFOR that nitazoxanide and more potent PFOR inhibitors have no effect in vitro [38].

Ref:

38. Bartelt LA, Bolick DT, Kolling GL, Stebbins E, Huston CD, Guerrant RL, et al. [Amixicile Reduces Severity of Cryptosporidiosis but Does Not Have In Vitro Activity against Cryptosporidium.](https://pubmed.ncbi.nlm.nih.gov/30297368/) Antimicrob Agents Chemother. 2018; 62(12):e00718-18.

10.  Line 348 re future studies of mechanism:  you should note also that it is unknown if these compounds are working by virtue of inhibiting Cryptosporidium factors or host factors.  There are many publications demonstrating dependence on host factors (e.g. protein kinase C (PMID 35099276) and aquaporin (PMID 15851691)).

**Response:**
Thank you for this suggestion. We agree with your comment. We added and modified this sentences in line 372-373, in our revised manuscript.

Our future studies will explore the mechanisms of these compounds against enzyme-related molecules because it is unknown if these compounds are working by virtue of inhibiting *Cryptosporidium* factors or host factors.

**Summary and General Comments**

**Reviewer #2**: This manuscript from Hazzaz Bin Kabir reports screening and initial in vitro and in vivo follow up of a collection of compounds based on traditional Chinese medicines against Cryptosporidium parvum.  These compounds haven't previously been screened against Cryptosporidium and several new leads with activity in a mouse model of infection were identified.  The methods used are well established and standard, and the work appears to have been executed well.  Overall, the results are of interest, as additional lead compounds are needed to develop better drugs for cryptosporidiosis.  The importance of the results is somewhat over-represented by use of the word "candidate" as far more work is needed before any of these compounds would be considered candidates, which implies a far more thorough evaluation regarding PK, dose-scaling, and toxicology.  That said, the compounds do represent interesting new leads worthy of further evaluation.  I have additional fairly minor
editorial suggestions as noted above.

**Response:**

Thank you for your comment. We agree with your concern. We replaced the word "candidate" and used the word “new drug leads” in the title and throughout our revised manuscript.

**Point-by-point responses to the Reviewer #3:**

**Methods**

**Reviewer #3:** For the analysis of the data in figure 4, a more appropriate statistical test would be repeated measures ANOVA (also available in GraphPad). This test compares the overall curves of each group rather than the data at different days. It is highly unlikely that there will be any difference in outcomes using this test, but it is just a better choice for the type of experiment performed.

**Response:** Thank you for your comments.

**Results
Reviewer #3:**  (No Response)

**Response:** Thanks.

**Editorial and Data Presentation Modifications?**

Reviewer #3: Lines 24-25 and 44 are oddly worded--the authors likely intended to state that cryptosporidiosis is the 2nd most common diarrheal disease, with rotavirus being the most common. But as worded, it sounds like cryptosporidiosis only occurs after someone has rotavirus infection.

The placement of a sentence about the anti-cancer effects of bufotalin in lines 77-78, between 2 sentences discussing compounds with anti-cryptosporidial activity is odd and at first gives the impression that bufotalin has previously been tested against cryptosporidia. It is probably best to move this sentence to the discussion.

Similarly, the sentence about bufotalin selectivity in lines 344-345 is oddly placed and breaks the flow of the paragraph. Perhaps the authors should create a separate paragraph discussing the effects of bufotalin in other studies.

**Response:** Thank you for this suggestion. We agree with your concern. We modified and revised this sentences in line 24-25, and 44 in our revised manuscript. We also moved the sentences about the anti-cancer effects of bufotalin in the discussion part in line 365-366, in our revised manuscript.

Moreover, we used a separate paragraph and revised this sentence in line 365-368, in our revised manuscript.

**Summary and General Comments**

**Reviewer #3:**  (No Response)

**Response:** Thanks.

**Point-by-point responses to the Reviewer #4:**

**Methods**

**Reviewer #4**: In the methods, this reviewer has the following concerns for the authors to consider:

1) The hit rate is quite high at 1 μM (9 out of 87 compounds), which is uncommon for a random collection of natural products. It is necessary to ensure that the phenotypic HTS assay used in the study is reliable.

For the in vitro screen and efficacy assays, fluorescence-imaging based quantification assay using Sporo-Glo (that also labels intracellularly developing C. parvum) is not one of the well-established assays. There is a need to show the quality of the assay, including: 1) standard curves (signal intensity vs. # of parasite inoculums) to show the linearity and linear dynamic range of the assay, and 2) dose-dependent curve of a standard control compound for comparison (e.g., NTZ or PRM).

There is also a need to describe how the fluorescence data were analyzed. Some representative images of the control and treated specimens will also be helpful in assessing the assay quality.
For the compound library, there is a need to give a brief description on the background of the 87-compound library, and citations if available.

**Response:**

Thank you for your critical comments. Our study was not a phenotypic HTS assay but we counted all the stained parasites by microscopy. Here we were checking the compounds dose dependent growth inhibition assay against parasites to make the standard curve as we are shown in figure 3. We have done dose-dependent curve of NTZ. The range of EC_50_ value of NTZ was 1.55-5.10 μM (PMID: 22167242, PMID: 29341586, PMID: 30297368), but our study found the EC_50_ value of NTZ 4.14±0.54 μM. Moreover, our main focus is to identify the potential new lead compounds in comparison to the positive control (parasite infected HCT-8 cells only).

For the fluorescence data analysis, we modified and added in line 153-156, in our revised manuscript.

Finally, cells were washed twice with 1 x PBS-T (1 x PBS supplemented with 0.01% Tween). A Keyence BZ-II 9000 was used to capture the images (KEYENCE, Osaka, Japan). The plates were imaged with a HS all-in-one fluorescence microscope using a 20x objective. The image output was imported into Microsoft excel for data organization and analyses. All C. parvum parasite in the well were counted.

For the background of compound library, we received these compounds from Toyama University, Japan. So far we know these TCMs groups have effective Chinese compounds. Each compound has each reference. However, there was no article, including all the compounds. That’s why all the references are huge.

2. In animal experiments using SCID mice:

A) Only female mice were used, and the oocyst shedding in SCID mice (max. at ~120 oocysts/gram of feces) was much lower than previous reported (typically in mg scale). The authors may want to add some discussion on the fact, and mention that there might be a need to further validate the in vivo efficacy in other models with both male and female animals.

**Response:**

Thank you for your comment. We agree with your concern. The oocyst shedding in SCID mice was lower in the control group. We are not sure, however, it may be the different number of oocysts used in different study for the infection of mice. Or maybe the choice of mouse model and parasite strains. We used SCID mice and HNJ-1 strain of *C. parvum* parasites, maybe other study used different mouse model or different strain of parasites. However, we think the gender of mice does not influence the oocyst shedding during infection as previously stated in the article (Qi et al., 2020).

Ref:

Qi L, Kogiso M, Du Y, Zhang H, Braun FK, Huang Y, et al. [Impact of SCID mouse gender on tumorigenicity, xenograft growth and drug-response in a large panel of orthotopic PDX models of pediatric brain tumors.](https://pubmed.ncbi.nlm.nih.gov/32891713/) Cancer Lett. 2020; 493:197-206.

B) Please clarify some important technical details, including: 1) how fecal pellets were collected from individual mice (e.g., all pellets dropped in past 24 h or fresh pellets dropped in the past 1 h); and 2) how oocysts were counted/quantified after sugar flotation method, plus critical details of the “sugar flotation method”.

**Response:**

Thank you for your comments. We took fresh fecal pellets from individual mice for counting of oocysts. The samples were examined for Cryptosporidium oocysts by using the centrifugal sugar flotation technique. Briefly, 1 g of fresh fecal sample was suspended in 9 ml of saturated sucrose solution (Specific gravity 1.266; 128g Sucrose and 100 ml distilled water) in a 15 ml tube, stirred, filtered through double wire-screen (50 and 100 mesh), and centrifuged at 1300 rpm for 5 min. Then, sucrose solution was added up to the 15-ml tube and stirred again which was then left at room temperature for 30 min. Then, the tube was filled up to the brim by adding drops of sucrose solution and putting a coverslip over the tube for 10 min. After that, the coverslip was put on a glass slide, examined, and counted for oocysts under a light microscope [Fujino et al., 2006]. We counted all of the oocysts in the whole area of coverslip observed under microscope.

Ref:

Fujino T, Matsuo T, Okada M, Matsui T. [Detection of a small number of Cryptosporidium parvum oocysts by sugar flotation and sugar centrifugation methods.](https://pubmed.ncbi.nlm.nih.gov/17146177/) J Vet Med Sci. 2006; 68(11):1191-3.

3. For the parasite strain (C. parvum strain HNJ-1): Please provide a GP60-based genotype/subtyping info (as an important parameter for comparison with other data in the literature).
**Response:**

Thank you for your comment. This *C. parvum* strain HNJ-1 was identified as GP60-based genotype IIaA15G2R1 (GenBank: AB237136) and had potential infectivity to human and other animals including cattle (Abe et al., 2006).

Ref:

Abe, N., Matsubayashi, M., Kimata, I. and Iseki, M. Subgenotype analysis of Cryptosporidium parvum isolates from humans and animals in Japan using the 60-kDa glycoprotein gene sequences. Parasitol. Res. 2006; 99: 303–305

**Results
Reviewer #4**: The results were reasonably well presented. Here are a few comments for clarification:

1. For clarity, it is suggested to change IC_50_ (recommended for use of 50% inhibition on a drug target in biochemical assays) to EC_50_ (recommended for use of 50% inhibition on parasite growth in vitro).

**Response:**

Thank you for your comment. We changed IC_50_ to EC_50_ throughout in our revised manuscript.

2. Histological images: Some local high-resolution images showing the parasites can be helpful.

**Response:**

Thank you for your comment. We added high-magnified images as insets (Fig 5E and 5F). There is no morphological changes in *C. parvum* oocyst between treated groups and non-treated (positive control) under light microscopic level, so we omitted to add similar insets for Figs. 5H, I, K, L, I, and O.

3. In Table 2: please define the parameter for the 95% CI (95% CI for EC50 or CC50 values?).
**Response:**

Thank you for your comment. In Table 2, the parameter of 95% CI is for EC_50_ values. We added this word in line 574, in our revised manuscript.

4. Primary screen data (anti-cryptosporidial activities in vitro of the 87 compounds at 1 μM) can be provided in spreadsheet as part of the supplementary materials.

**Response:**

Thank you for your comment. We provided the primary screen data in a spreadsheet as part of the supplementary materials and mentioned in line 622-624, in our revised manuscript.

**Editorial and Data Presentation Modifications?**

**Reviewer #4**: 1) In reviewing anti-cryptosporidial drug discovery, there are several other good review articles and original research articles reporting phenotypic-based HTS. A more relevant one is the HTS of natural products (PubMed PMID: 31551955; PubMed Central PMCID: PMC6736568).

**Response:**

Thank you for your suggestion.

2. Line 23, Abstract, first sentence: “Cryptosporidium spp. are intestinal opportunistic protozoan parasites…”: change intestinal to gastrointestinal (as there are also gastric Cryptosporidium species such as C. muris).

**Response:**
Thank you for this suggestion. We changed and revised this word in line 23, abstract in our revised manuscript.

3. Other language and grammatical issues:

Line 46 and many other places:  Please make “Cryptosporidium” (anti-Cryptosporidium) and “C. parvum” in italic.

Line 67 and other places if any:  change the first letter in “Nitazoxanide” to lowercase (nitazoxanide) unless it starts a sentence. The same applies to other compound names and terms such as “Paromomycin”, “Malaria”, “Toxoplasmosis” and “Cancer” (lines 89 -90).

Some other irregular usages. For example, leave a space between a number and unit (e.g., line 164, 100μl could be 100 μL; line 172, the μ in μM does not need to be in italics …).

**Response:**
Thank you for this suggestion. We agree with your comment. We made this word “*Cryptosporidium*” (anti-*Cryptosporidium*) and “*C. parvum*” in italic throughout in our revised manuscript. We also changed the first letter in “Nitazoxanide” to lowercase and other compound names in line 68, 83, 89, and 90 in our revised manuscript. Moreover, we put a space in between a number and unit in line 171, 179, and throughout in our revised manuscript.

**Summary and General Comments**

**Reviewer #4**: In this manuscript, Bin Kabir et al. reported the identification of anti-cryptosporidium activity of four compounds from traditional Chinese medicine herbs in vitro and in vivo (SCID mice), i.e., alisol-A, alisol-B, atropine sulfate and bufotalin. The four hits showed excellent, nanomolar anti-cryptosporidial activities (EC_50_ = ~80 to ~250 μM). Two compounds (atropine sulfate and bufotalin) were further evaluated in vivo, showing efficacy against the infection of C. parvum (reduction of oocyst shedding).

These observations are very interesting and important by providing new structures for potentially further development of anti-cryptosporidial therapeutics.

**Response:**
Thank you for your appreciation.
